# Arthropods as Models for Transdisciplinary Bio-Inspired Research and Discovery

**DOI:** 10.3390/insects17020165

**Published:** 2026-02-02

**Authors:** Jillian Kurovski, Nicole R. Sexton, Angela K. Pannier, Eileen A. Hebets

**Affiliations:** 1 School of Biological Science, University of Nebraska-Lincoln, Lincoln, NE 68588, USA; jkurovski2@nebraska.edu (J.K.); nicole.sexton@unl.edu (N.R.S.); 2 Biological Systems Engineering, University of Nebraska-Lincoln, Lincoln, NE 68583, USA; apannier2@unl.edu

**Keywords:** biomining, climate change, health, materials innovation, organismal biology

## Abstract

This opinion article proposes that arthropods, with their tremendous biodiversity and evolutionary resilience, can offer abundant opportunities for integrative, transdisciplinary, solution-focused research. To support this proposal, we first (1) introduce arthropods and showcase their deep evolutionary history and tremendous diversity. Next, we (2) briefly discuss the role of arthropods in the history of human innovation and highlight some of the challenges they have helped us overcome. We provide select examples of the importance and innovation of arthropods in food, medicine, agriculture, materials, and technology. We then discuss (3) arthropods and grand challenges, articulating how they are both part of the problem and key to the solution. (4) We discuss multiple models for creating transdisciplinary research teams—1. challenge-focused, 2. taxon-focused, and 3. innovatively open-ended with respect to challenge and taxon—and the scales at which they can be created—local/regional/global. Finally, (5) we suggest that arthropods can provide a centering focal point for transdisciplinary research; an inspirational magnet that can pull together distinct scholars to coalesce and collaborate around a common nature-based theme. Our strategic next steps include exploring open-ended arthropod-focused research teams, as they would be well-positioned to tackle multiple solution-based collaborations, and such collaborations are likely to lead to novel discoveries and nature-inspired innovations.

## Simple Summary

In this opinion article, we propose that arthropods—insects, crustaceans, centipedes and millipedes, and arachnids—can act as a unifying theme to bring together researchers with distinct expertise to solve current challenges. Following a brief introduction of arthropods that highlights their deep evolutionary history and tremendous diversity, we provide an overview of the role of arthropods in the history of human innovation, including examples of arthropods in food, medicine, agriculture, materials, and technology. Next, we discuss how arthropods are part of our largest challenges but may also be key to the solutions. We discuss multiple models for creating transdisciplinary research teams—(1) challenge-focused, (2) taxon-focused, and (3) innovatively open-ended with respect to challenge and taxon—and the scales at which they can be created—local/regional/global. We then focus our arguments on the third model, suggesting that arthropods can provide a centering focal point for transdisciplinary research, and we propose that they act as an inspirational magnet to pull together distinct scholars to coalesce and collaborate around a common nature-based theme. Ultimately, we encourage more open-ended teams, concluding that a transdisciplinary research community centered generally on arthropods would be well-positioned to tackle multiple solution-based collaborations, and that such collaborations are likely to lead to novel discoveries and nature-inspired innovations.

## 1. Introduction

Living arthropods—comprising Chelicerata, Myriapoda, and Pancrustacea [1,2], which include insects, crustaceans, centipedes and millipedes, and arachnids—encompass a diverse and resilient group of animals that have survived on our planet for hundreds of millions of years, living in virtually all environments. Although they represent only one of the more than 30 extant animal phyla [2], with more than 1.5 million described species, arthropods make up around 80% of all known animals [3]. Recent estimates suggest that there could be as many as 7 million arthropod species [4]. It is perhaps not surprising, then, that with their abundance and adaptability, arthropods have a deep, intertwined history with human society.

From the formation of the Silk Road by the humble silk moth to cockroach-inspired search and rescue robots [5,6], arthropods have been central to human innovation and progress. Simultaneously, arthropods have posed (and continue to pose) some of humankind’s most pressing challenges, from insect pests that devastate our crops and reduce our food supply to arthropod disease vectors (e.g., mosquitoes and ticks) that carry and transmit deadly pathogens. Even beyond arthropod-derived material inspiration and health risks, our lives are interdependent on our arthropod relatives in innumerable ways. The ecosystem services provided by arthropods, and the bioinspiration derived from them [7,8], touch nearly every aspect of society, including health, agriculture, engineering, biological pest control, and culture [7,8,9,10].

In this opinion article, we first highlight some important contributions that arthropods have made to human society and innovation. We next articulate current challenges facing humankind. Finally, we argue that with their history of success and survival, arthropods provide a deep reservoir of knowledge from which to draw upon for future solutions to grand challenges. We conclude that arthropods are an ideal focal point for transdisciplinary research: research that seeks to understand and influence broad-scale change through transdisciplinary collaboration. Ultimately, we encourage scientists across disciplines to consider arthropods as a shared model system through which transdisciplinary collaboration can spark innovation.

## 2. A Brief Glance at Arthropod Importance and Innovation

Drawing from nearly 500 million years of arthropod evolution, humans have been able to continuously derive creativity and innovation from arthropod biology. Arthropods have been used as models for problem-solving, engineering, and artistic inspiration. They have shaped our culture and society from medicine to architecture, food, and technology. Arthropods have been instrumental in major breakthroughs, including the Silk Road that vitalized world trade, founded by the silk moth [5], or Shellac, sourced from the secretions of the small (2 mm-long) female lac bug, which is used to coat and protect the finest instruments, wooden furniture, homes, and artwork [11]. Beyond innovations, arthropods have provided us with a foundational understanding of genetics, neurobiology, and behavior (e.g., research on the fruit fly [12]). We owe many of our greatest successes to arthropods, including our continued survival.

In this first section, we provide some relevant examples of the historical role of arthropods in (i) food, (ii) medicine, (iii) agriculture, (iv) materials, and (v) technology. We note that we have chosen only select examples to highlight, as an inclusive and complete review of the history of arthropod importance and innovation is beyond the scope of this opinion article.

### 2.1. Arthropods and Food

Arthropods have long been a part of the human diet [13,14,15] and likely played an important nutritional role for early humans, providing a low-capture-risk source of protein and important fatty acids and lipids [16,17]. There is evidence that arthropods in early human diets have included termites [15], grasshoppers [18], and crustaceans [19,20]—arthropods that are still consumed by humans today. Historical and more modern contexts demonstrate how entomophagy (the practice of eating insects) intersects with cultural identity and themes of human resilience. In South Korea, silkworm pupa *Bombyx mori* (Linneaus 1758), known as beondegi 번데기, became a cheap source of protein during the post Korean war era [21]; and in Japan during World War II, food scarcity led to silk mill workers eating silkworm pupae, known as kaiko カイコ [22]. Chapulines—Mexican grasshoppers that are emblematic of Oaxaca’s indigenous roots—served as important sources of protein during the seventeenth and eighteenth centuries, and even during the COVID-19 pandemic [23]. Entomophagy remains a cultural tradition across the globe, with an estimated several hundred million people consuming [24] the >2000 recorded edible insect species [25]. Similarly, consumption of crustaceans remains widespread. Canada is the largest producer of snow crab, at 90,000 tons reported in 2025 [26]. China is the largest producer of red crab, with total crab production near one million tons in 2025 [26].

Entomophagy extends beyond whole animal consumption to its influence on food products like honey and figs. These products rely on arthropods, and both have a deep history and a current global market. One of the oldest human records of honey dates to 20,000 years ago, where wall paintings depict honey harvesting in modern-day Zimbabwe, South Africa, and Zambia [27,28]. Similarly, cave paintings from Europe at the end of the last glaciation, 20,000 to 13,000 years ago, show a collection of honey via rope ladders [17]. Today, around 94 million beehives worldwide produce about 1.77 million tons of honey each year [29]. Like how honey requires bees, some varieties of figs require wasp pollination (e.g., Calimyrna figs) [30]. Overall, pollinators account for 5–8% of global crop production [31], and non-bee insects account for 25–50% of the floral visits of globally important crops [32].

Arthropods are also increasingly used in animal feed for livestock, pets, and captive animals as a replacement for traditional protein sources such as poultry or pork [33,34,35]. Arthropod substitutions come in various forms, such as flours, oils, or whole insects [34]. The use of arthropods in animal feed has been shown to reduce pathogen presence in livestock [36] and their manure [37], encourage weight gain [38], and promote sustainable farming practices, offering improvement in global food security [16,33,34,39].

### 2.2. Arthropods and Medicine

From ancient to modern times, arthropods have been closely linked with human health. While often considered as carriers of disease (e.g., fleas and the bubonic plague, mosquitoes and malaria, and ticks and Lyme disease), arthropods have also served as medicine and a source for biomedical innovation. Silk, for example, is one of the oldest arthropod-derived biomedical materials, with a history that can be traced back nearly 7000 years [40]. Ancient Greeks and Romans bundled unprocessed spider silk to treat wounds [40], and more recently (1800s) used it as sutures for incisions [41]. Since then, spider silk has been investigated for its potential in tissue engineering [42,43,44] due to its high biocompatibility, low immunogenicity, limited bacterial adhesion, and controllable biodegradability [40,45].

Arthropod antibacterial peptides (cockroaches [46]) and affinities for dead tissues (maggots [47]) offer possibilities for the development of novel antimicrobials and targeted wound cleaning processes, respectively. Horseshoe crabs are perhaps most infamous for the production of limulus amebocyte lysate (LAL) in their bright blue blood [48]. The LAL assay is widely used to test vaccines for contamination, checking for bacteria [49].

Arthropod venom also offers tremendous pharmaceutical potential. Bee venoms (apitoxin) have anti-inflammatory, anti-arthritic, and neuroprotective properties against Parkinson’s and Alzheimer’s disease [50]; wasp and hornet venoms contain anti-inflammatory and anti-microbial properties [51,52,53]; while scorpion, centipede, and spider venom have been identified as containing novel compounds with painkiller potential [54]. Further, a scorpion-derived venom protein can make brain tumors fluoresce [55,56], aiding in the precise identification of cancerous tissue.

Arthropods are not only sources of important biomedical resources, but they often serve as stand-in models for testing new and innovative pharmaceuticals. The fruit fly, *Drosophila*, has long been used as a model for human disease, as it shares ~60% of its genes with humans [57]. Additionally, 62% of human disease genes are conserved in *Drosophila* [58]. With a complete genome [59,60] and a complete connectome [61], the opportunities afforded by *Drosophila* to study health and disease range from cancers and hereditary diseases [57] to the impacts of microplastics on human health [62].

Ultimately, arthropods hold a dual role in human health as carriers of disease and sources of healing. Their unique properties have resulted in new therapies, drugs, and methods of care that span from closing wounds to cancer treatment. Despite significant advances, there remain numerous unrealized opportunities for arthropods in medicine.

### 2.3. Arthropods and Agriculture

Like in human health, arthropods have long played a dual role in agriculture—as beneficial organisms but also as persistent pests. Positively, they serve directly as pollinators and predators to herbivores [31,32,63,64]. Arthropods such as spiders [65,66,67], ladybugs [68,69], mantids [70], predatory mites [71], and parasitoid wasps [72] have also been investigated for their use as biological control agents.

Arthropods are an estimated 85% of all soil-dwelling fauna and play a central role in nutrient cycling and crop productivity [73]. They indirectly exert a positive force through their contributions to soil health. Negatively, the age-old struggle against crop damage has continually shaped human innovation from the past to the present. Evidence of negative insect impacts on early human societies can be traced back to the Ancient Sumerians, who used sulfur compounds to control insects and mites, and the Greek poet Homer referenced burning as a strategy for locust management [74]. These struggles predate the expansion of human agriculture (~4200 BCE [75]) and remain major challenges today.

Advances in both technology- and arthropod-based solutions show promise for combating insect pests. Bisgin et al. (2022) [76], for example, developed an AI algorithm capable of identifying food-contaminating beetles, enabling more accurate risk assessment and targeted management strategies. Modern insecticides increasingly emphasize environmental sustainability and allow for more controlled release and reduced ecological impact [77,78]. On the arthropod-solution side, Barragán-Fonseca et al. (2022) [79] demonstrated that incorporating black soldier fly, *Hermetia illucens* (Linnaeus, 1758), exuviae into soil increased plant biomass, flower number, pollinator attraction, and seed yield, while also enhancing tolerance to herbivory. Beyond agriculture, black soldier flies, along with crickets, mealworms, grasshoppers, and house fly maggots, are also increasingly recognized as sustainable sources of animal feed, providing essential nutrition while reducing the need for high-resource-demand livestock feed such as soybeans [80,81]. From ancient pest management practices to cutting-edge technologies and sustainable food innovations, arthropods remain central to agricultural challenges and solutions alike.

### 2.4. Arthropods and Materials

Many of our finest materials are thanks to arthropods. Silk, for example, has long been leveraged for applications from luxury textiles to industrial packaging [79], parachutes, and even tissue repair (see also *Arthropods and Medicine*) [80]. Spider silk continues to attract researchers because of its strength, flexibility, durability, humidity response, light transmission, thermal conductance, and shape memory [82], and modern advances in textiles continue to utilize these properties [83,84]. Arthropod exoskeletons are also known for being tough and rigid, yet lightweight and durable. Researchers are even exploring sustainable bioplastics based on arthropod exoskeletons, which could be useful for biodegradable packaging, injectable fillers, 3D printing ink, wound dressing, and implantable devices [85].

The fine structures of arthropods have also led to advances in optics and nanomaterials. Butterfly wing scales are made of hyperfine structures to reflect vivid colors that attract mates [86], ward off predators [87,88], and aid in camouflage [89]. These nanostructures are models for research into optical detection through refractivity and could be applied to water-quality monitoring and analysis [90]. The structure of blue morpho butterfly wings (*Morpho Menelaus*) has also inspired new infrared detection technology [91], as well as vapor sensors with applications towards high-performance gas separators and photonic security tags [92]. Similarly, beetle iridescence has also been studied for its colorful and strength-based properties, resulting in biomimetic materials [93] that can be applied to durable coatings [94], buildings [95], and adhesives [96,97,98]. The sound production organ of cicadas has even been an inspiration for novel designs of acoustic transducers [99]. Ultimately, arthropods have given rise to many breakthroughs in material science [100], offering nature-based design principles with a multitude of examples yet to be explored.

### 2.5. Arthropods and Technology

Arthropods have both an ancient and modern history of inspiring human tools and technology. From flight and locomotory mechanics to sensory systems, arthropods provide natural blueprints for modern advances in technology. Drone technology, for example, has improved its aerodynamic performance by mimicking the kinetics of wing flapping in 3D space [101], while the Moroccan flic-flac spider (*Cebrennus rechenbergi* (Jäger 2014) [102]) serves as a model of locomotion over sand and snow with its rapid cartwheels down sand dunes [103]. Similarly, the cockroach’s prowess at moving through tight spaces has inspired the development of search-and-rescue applications following natural disasters through robots that mimic cockroach locomotion [6]. Insect neuronal networks are even being studied and mimicked with the goal of improving processing capacity at small scales and integrating novel AI strategies into autonomous robots [104].

Arthropod sensory systems are diverse and often times highly specialized, providing a wealth of biodesign blueprints. Cameras with extreme depth have been inspired by the compound eye of extinct trilobites [105], while jumping spider eyes inspired endoscope dual cameras with a wide-angle field of view and increased resolution [106]. Furthermore, insect compound eyes have inspired motion sensors for autonomous vehicles to detect surroundings [107]. With olfaction, scientists have employed AI to determine the neural patterns associated with odor sensing to be used in narcotic detection and disease biomarkers [108], while a sensor inspired by selective insect olfaction is being explored for a function in detecting human disease (through breath) [109]. Like the other categories above, numerous opportunities exist for additional arthropod-inspired technological innovations that can address grand challenges.

## 3. Arthropods in Grand Challenges

Arthropods have weathered more than 500 million years of Earth’s grand challenges—from mass extinctions to climate upheavals—emerging each time with novel adaptations that allow them to manipulate their environments, outcompete rivals, and radiate into nearly every ecological niche. Throughout human history, they have played critical roles in helping us overcome our own challenges—from pollination and decomposition to inspiring medical and materials innovations. Their resilience, diversity, and biological ingenuity make them ideal partners—and platforms—for developing solutions to today’s pressing local and global challenges in human and environmental health.

Grand challenges are some of the world’s most difficult-to-solve problems, spanning cultures and continents, distinguished from traditional research questions by their complexity, emergent properties, nonlinear dynamics, and numerous interactions and interconnections [110,111]. Commonly identified modern grand challenges include climate change and environmental sustainability; global health and pandemics; poverty, inequality, and social justice; ethical technological advances (e.g., artificial intelligence); energy security; food and water security; global governance, peace, and safety; education, workforce development, and knowledge access; and/or infrastructure and urban resilience.

In human health, arthropods are responsible for transmitting a variety of pathogens that result in severe morbidity and mortality in humans and agricultural animals [112]. Mosquitoes alone transmit pathogens that are collectively responsible for nearly one million deaths worldwide every year, and most deaths are concentrated in developing nations and impoverished communities [113]. Seven of the Gates Foundation Global Grand Challenges center on mosquitoes, with an emphasis on preventing and eradicating malaria [114]. The Entomological Society of America [115] and Royal Entomological Society [116] both list combatting vector-borne diseases as a grand challenge. The Institute of Electrical and Electronics Engineers (IEEE) specifically lists the detection of mosquito breeding grounds among its grand challenges [117].

Food security is another grand challenge in which arthropods play a critical dual role. As pests, invasive insects cost the United States US 126.42 billion annually, with most of the cost associated with agriculture [118]. As beneficial animals, insects are increasingly seen as a potential sustainable solution to global hunger and to reduce emissions from traditional meat production [119], which accounts for approximately 15% of all anthropogenic greenhouse gas emissions [120]. More than 2200 species of insects—including grasshoppers, beetles, and ants—have been identified as edible for both humans and livestock [25]. Organizations and institutions such as the University of California Davis, Cornell University, the National Institutes of Health, and the American Society for Animal Science list food security amongst their targeted grand challenges, citing the role of arthropods as agricultural pests as well as potential sustainable solutions as food for both humans and animals.

Arthropods have always been intertwined with human innovation and addressing humanity’s grand challenges, but their potential for influence may soon be overlooked. As we have demonstrated, humans have a history of overcoming challenges with bioinspired solutions—solutions inspired by biological structures or processes [121]. Yet, as the need for innovative solutions grows, we face a time of unprecedented biodiversity loss [122,123] and a decline in human connection with nature [124]. In reducing our connection with nature, we risk suffering an “extinction of experience” whereby we become increasingly unfamiliar with the natural world [125,126,127]. Furthermore, in the throes of the Anthropocene, arthropods in particular are facing a significant and concerning decline [128,129]—a loss even non-scholars have noticed, sometimes dubbed the ‘windshield phenomenon’ [130,131], which is a poignant nod to drivers feeling there are fewer insects on their windshield after traveling. As species are confronted with decline and extinction, coupled with an increasing human disconnect from the natural world, the devastating result is that we risk overlooking arthropods not only as ecological keystones, but also as powerful sources of solution-based biomimicry and biodesign.

The complexity of grand challenges dictates that no single discipline can meaningfully provide solutions [110,132,133,134]. Critically, leveraging arthropods for bioinspired research and discovery requires an integrated group of individuals encompassing distinct and diverse areas of expertise, experiences, and outlooks. Solving grand challenges means acknowledging and simultaneously addressing the complexity and interconnected nature of the problems—including the social and environmental factors (e.g., social support, politics), individual factors (e.g., a single person’s risk factors), and biological factors (e.g., physiological pathways, genetic factors [135]). Grand solutions require dismantling traditional academic silos and building a transdisciplinary approach that fosters collaboration across scientific, technological, social, and ecological domains. It requires an integrative approach to problem-solving that extends beyond disciplinary boundaries to co-create new knowledge and new solutions. We define transdisciplinary as academic and non-academic (e.g., community members, policy makers, and industry experts) collaborators operating beyond their respective disciplines for formulating shared questions and working within a shared framework [135,136,137]. Such a transdisciplinary approach is not easily achieved, as it requires not only a willingness to engage with different kinds of expertise but also a shared framework that can serve as a common ground for inquiry, experimentation, and innovation. Transdisciplinary research is more than aligning around a shared problem—it is about realizing a collective vision through a shared framework, where diverse expertise integrates to address complex questions.

## 4. Models for Developing Arthropod-Focused Transdisciplinary Research Innovation

The application of arthropods to solve grand challenges is boundless, but building transdisciplinary research teams is challenging. Our currently siloed research and education institutions make it difficult to connect scholars from distinct areas of expertise. While many researchers may be interested in collaboration across disciplines, such collaboration is hindered by departmental silos, cultural divides, and discipline-specific jargon and methodology [138,139,140]. Nonetheless, networking, expansive funding calls, and grassroots collaboration efforts, such as facilitated brainstorming workshops, can help build cross-disciplinary bridges and collaborative teams.

We conceptualize three main models of arthropod-focused research teams: (1) those built around a focal, pressing challenge—e.g., vector-borne disease or food insecurity; (2) those built around one focal group of organisms (e.g., spiders and their silk); or (3) those more fluid with respect to challenges and focal organisms (e.g., encompassing arthropods and challenges broadly). Furthermore, these teams can form at multiple scales—e.g., within an institution or community, encompassing multiple institutions/communities within a geographic region, and/or internationally/globally.

While the type of arthropod-focused research team (i.e., solution-based and challenge-focused, taxon-focused, or inclusive of multiple taxa and challenges) and scale of the team (local, regional, or global) may vary, they all require the same foundation. They need researchers and community members who are (i) open to new ideas, (ii) willing to expand their work beyond their comfort zone, and most importantly, (iii) open to, and excited by, collaboration.

### 4.1. Challenge-Focused Research Teams

Many topic- or challenge-specific research teams have already built successful, federally funded collaborations around arthropods. The Center for Vector-Borne Infectious Disease at Colorado State, for example, focuses on infectious disease, while the University of Florida and the University of Kentucky’s Center for Arthropod Management Technologies bring together university researchers, government, and industry partners to find solutions for arthropod pests of agricultural, medical, and veterinary importance. The Center for Environmental Sustainability through Insect Farming is composed of faculty from Texas A&M, Indiana University–Purdue, and Mississippi State University, with a focus on developing methods for using insects as feed for livestock, poultry, and aquaculture. Region-specific teams include the International Center of Insect Physiology and Ecology at the University of Nairobi. The mission of this latter center is to reduce poverty, ensure food security, and improve the health of people of the tropics through local and international collaboration aimed at developing management tools for harmful and useful arthropods. These established and impactful arthropod research centers provide a strong foundation for transforming how communities approach grand challenges. Notably, in many instances, federal funding calls have facilitated the formation of these research centers. We both advise and hope for the continuation and even expansion of such forward-thinking funding opportunities.

### 4.2. Taxon-Focused Research Teams

Instead of focusing on specific challenges, a specific taxon-centered research team may explore challenges and innovations through a focal organism or group of organisms. A focus on spiders, for example, could explore biomedical innovations associated with both venom and silk, while simultaneously investigating spiders’ roles in ecosystem services [7]. Similarly, black soldier flies could provide research opportunities in waste management, sustainable animal feed, bio-based material innovation, soil health and carbon sequestration, human and animal health, and genetic engineering. Research groups such as the Swedish University of Agricultural Sciences Honey Bee Research Center use this taxon-centered approach to conduct research on environmental health and outreach through honeybees. Again, funding opportunities to support broad taxon-focused research are imperative for associated bioinspired discovery and innovation.

### 4.3. Open-Ended Research Teams

Finally, a more fluid research group may unite over the simple idea of arthropods (inclusive of any focal taxon) as inspiration for novel solutions to multiple local/global challenges and scientific advances. Such an innovative open-ended approach to team building offers limitless opportunities for community buy-in. For example, biomedical engineers working on innovating biomaterials and nucleic acid delivery systems to advance gene and cell therapies or vaccine strategies might be inspired toward innovative design solutions from their collaborations with researchers studying mosquito–virus systems. Or chemists working on biosensors for environmental contaminants might find inspiration from collaborators researching chemical receptors present in hymenopterans. Meanwhile, entomologists investigating potential sensory-related deterrents of agricultural pests can be inspired by an arachnologist studying sensory modalities used in spider courtship. We propose that this third approach represents a pioneering method to form teams, to share knowledge, and to address grand challenges by creating a community that studies multiple taxa through a variety of expertise and skillset lenses, allowing siloed disciplines to converge, integrate, and brainstorm. We expand on these ideas in the next section.

## 5. Arthropods and Transdisciplinary Research

Transdisciplinary teams that unite broadly around arthropods, without a defined challenge or goal (i.e., open-ended research teams), offer numerous opportunities for scientific advances through the unique communities they can build. First, despite being broad, such nature-based unification still provides both a reason to come together and a source of solution-based inspiration. It ensures that research and ideation are centered on a common ground that enables mutual learning, innovation, and synergistic discovery. Second, a shared system acts as a living skeleton supporting different perspectives—whether ecological, economical, biological, mechanical, behavioral, or artistic—that can interact productively and iteratively [141,142]. Coalescing around arthropods reduces barriers to collaboration by providing a common theme and a shared structure for inquiry. Third, an open-ended approach widens the net for capturing different methodologies and questions.

Notably, arthropods are not just a bridge for connecting disciplines, but a means of connecting cultures, and thus, connecting collaborators on a more holistic level. Arthropods inspire public fascination, facilitating accessible education in multiple settings, including formal classrooms, museums, and community science projects (such as iNaturalist). The ecological, cultural, and economic impacts of arthropods set a strong foundation for buy-in across collaborators. From bioindicators [9] and pollinators [31,32] to textiles [83], wax, food [25], and medicine [42,52,56], inspiration for architecture [95], machines, materials [100], and art [11], every person is touched by arthropods in their daily lives [11]. Every model organism comes with benefits and disadvantages, but arthropods’ combination of diversity and cultural relevance is difficult to match. They provide a natural bridge between disciplines and cultures, offering collaborators a shared point of reference that is both scientifically rich and socially meaningful.

Using arthropods broadly as a centering point provides innumerable paths to appeal to and recruit different researchers and community members, whether drawn in by their personal interests (e.g., a love of butterflies or a family member with Lyme disease) and/or specific areas of expertise (e.g., robotics). It increases the likelihood of buy-in from a diverse group of individuals to provide new perspectives, affords almost limitless ways to engage within academic and local communities, and increases the chances for leadership roles and/or influencing research directions for each team member. Diversifying and expanding the team increases available knowledge (organismal and more), skillsets, and toolkits available for each system and individual researchers. A broad arthropod-focused research and education community also increases the likelihood that any knowledge gains, technological advances, or other breakthroughs in one system can advance research in another, whether through direct methodology transfers or by igniting curiosity and innovation in another system.

Leveraging arthropod biology and resilience for bioinspired breakthroughs, innovation, and problem-solving necessitates new knowledge gain, as it minimally requires (a) taxon-specific information about arthropod ecology and behavior; (b) identification, development, and testing of arthropod-derived or -inspired materials; (c) innovations in husbandry and large-scale production of arthropods; and (d) the intersection of arthropods and humanity through education, community engagement, and art. Meeting these requirements lends to the necessity of more than isolated projects. It will require, for example, engineers to learn from arachnologists about the numerous distinct types of spider silk and their many natural functions, or virologists to learn from entomologists about mating or feeding behavior in arthropod vectors. In today’s expansive and siloed research enterprise, we propose that major future advances and innovations will require true transdisciplinary teamwork. It will call for a community of researchers connected through a shared framework that is inspired and guided by one of nature’s most amazing groups of animals—arthropods.

## 6. Summary and Strategic Next Steps

Arthropods—with their immense biodiversity, ecological ubiquity, evolutionary resilience, and application across disciplines—offer abundant opportunities for integrative research. Arthropods inhabit nearly every ecosystem on Earth, often in high densities, and their diverse morphologies and life histories present both challenges and opportunities for humanity. Despite the evolutionary problem-solving of arthropods that allows them to thrive in a wide range of environments, arthropods remain underutilized in efforts to discover novel solutions to global challenges through a transdisciplinary framework. We argue that open-ended research teams can, and should, serve as a powerful model system for uniting diverse groups of researchers to address pressing issues in health, sustainability, technology, and beyond. Grounding collaboration in this shared biological framework can foster interdisciplinary connections, inspire innovative research, and support the development of holistic, scalable, and equitable solutions.

A transdisciplinary research community with comprehensive integration of research, educational programs, and community-building approaches centered on arthropods would be well-positioned to undertake solution-oriented collaborations. Such a community could encompass a spectrum of arthropod-related disciplines, including vector-borne disease, agriculture, environmental health, nutrition, robotics, sensors, arts, education, and materials innovation, and organically extend to community-building approaches, including mentorship models, cross-sector partnerships, and outreach programs led by and for local community members. The arthropod-centered transdisciplinary community could act as a hub for cutting-edge research while simultaneously being a vibrant connector that engages students, professionals, and the public through symposia, workshops, and educational courses. This nexus would purposefully align arthropod-related research with human and environmental health, feeding directly into a pipeline for innovation in arthropod-derived and use-inspired materials innovation.

While this paper focuses on arthropods, the proposed transdisciplinary framework built around a natural model system is broadly applicable. Other resilient and diverse taxa—such as fungi, coral reefs, or cephalopods—hold great potential as unifying systems for transdisciplinary research. These organisms, like arthropods, span ecological, evolutionary, and technological relevance, offering rich opportunities to address complex global challenges through integrated inquiry. Thus, we encourage researchers across all disciplines to reach beyond comfort and meaningfully engage with their academic and local communities through a central model system, integrating and expanding each other’s knowledge and skill sets to confront humanity’s greatest challenges.

## Data Availability

No new data were created or analyzed in this study. Data sharing is not applicable to this article.
